# Ideal family size decision and its associated factors among women of reproductive age: community survey in southern Ethiopia

**DOI:** 10.7717/peerj.15103

**Published:** 2023-03-21

**Authors:** Misganu Endriyas, Agegnehu Gebru, Amare Assefa

**Affiliations:** 1 SNNPR State Health Bureau, Hawassa, Sidama, Ethiopia; 2 Transform Primary Health Care Project, Hawassa, Sidama, Ethiopia

**Keywords:** Contraceptive utilization, Counselling, Family planning, Ideal family size, Fertility preferences

## Abstract

**Background:**

Understanding fertility preferences is important for population studies and planning programs. The ideal family size, which is the number of children wanted in one’s lifetime, is one of variables used to measure fertility preferences. However, there was limited information on ideal family size decision in Southern Ethiopia. Therefore, this study was designed to assess ideal family size decision and its associated factors among women of reproductive age in Southern Ethiopia.

**Methods:**

A community based cross-sectional study was conducted in 2015 in Southern Ethiopia. Multi-stage stratified cluster sampling was used to select 3,205 study subjects. Data on socio-demographic characteristics, reproductive history, deciding ideal number of children, knowledge and utilization of contraceptives were considered. Descriptive statistics and binary logistic regression were done to describe and assess factors associated with deciding ideal family size. The association between variables was presented using odds ratios with 95% confidence intervals.

**Results:**

We included 3,205 women of reproductive age from which 37.5% respondents could not read and write and 56.5% were housewives. About half of the respondents, 47.1%, did not decide ideal numeric family size or failed to report numeric preferences while 21.6% desire to have five or more children. The mean ideal number of children preferred was 4.5 ± 1.62. Educational status (*P* < 0.001), overall knowledge about contraceptives (*P* < 0.001), current contraceptive use (*P* < 0.001), place of residence (*P* < 0.001), age (*P* < 0.004), marital status (*P* < 0.003) and number of living children (*P* < 0.003) were factors associated with deciding ideal family size.

**Conclusion:**

Only about half of respondents decided ideal family size from which one fifth prefer high fertility. The mean ideal number of children was comparable with that of Sub-Saharan estimate. Counselling that can empower women to decide family size should be strengthened to empower less empowered women.

## Background

The fertility preferences of women refers to women’s desired family size and intention to limit or delay childbearing (Sarnak et al., 2021). The ideal family size, or number of children wanted in one’s lifetime, is one of variables used to measure fertility preferences or for estimating the level of wanted and unwanted fertility (Thomson, 2015).

Although fertility has been declining in developed countries (Skakkebæk et al., 2022), evidences show that the desired family size is relatively high in Sub-Saharan Africa (Atake & Gnakou Ali, 2019; Matovu et al., 2017). This high fertility has negative impacts on the health of children and their mothers, child education, human capital investment, economic growth and environment (Pinter et al., 2016; World Bank, 2010; Forson, Arthur & Ayeh-Kumi, 2018; Blaabaek, Jaeger & Molitoris, 2020).

Economic theories of childbearing relate decisions about family size and the timing of births with the constraints on incomes and prices (Ermisch, 2001). In low- and middle-income countries, decisions about a women’s fertility is influenced by several factors like women empowerment, partner influences, social norms and cultural context (Haque et al., 2021). On the other hand, perceived ideal number of children is associated with residence, religion, employment and experience of child death (Akram et al., 2020).

Understanding women’s fertility preferences is important for monitoring population growth and developing policies and programs like family planning (FP) (The DHS Program, 2007). However, there was limited information on ideal family size decision in the study setting, Southern Ethiopia. Therefore, this study was done to determine decision on ideal family size and its associated factors among women of reproductive age in Southern Ethiopia.

## Methods

### Study design and setting

A cross-sectional study was conducted in Southern Nations Nationalities and People’s Region (SNNPR) of Ethiopia, which is the third largest administrative region of the country representing about 20% of the country’s population at the time of data collection. It is the most diverse region in the country in terms of language, culture and ethnic background (UNICEF Ethiopia, 2021). Currently, the region is administratively sub-divided into three regions: Sidama, SNNPR and Southwest regions.

### Data source, sample size and sampling

The data used for this study was first collected to assess contraceptive utilization and its associated factors in SNNPR Ethiopia in 2015 (Endriyas et al., 2017). It was a community-based cross-sectional survey. The region was stratified into urban, agrarian and pastoralist to represent regional variability of contraceptive utilization. Multi-stage sampling was used to select study participants. First, 20% of woreda were selected from each stratum. Woreda, equivalent to districts, is administrative structure with approximate population of 100,000. Based on this assumption, five towns, 33 agrarian and two pastoralist woredas, a total of 40 woredas were selected. Second, two kebeles (smallest administrative structures) were selected from each woreda. Third, two health development networks were selected from each kebele. Health development networks (locally called limat budin) are networks of 25–30 households in which one leader leads 25–30 households towards good health practices. Finally, 3,205 women in reproductive age group (15–49 years) were included in the study using cluster sampling.

### Variables

Data on socio-demographic characteristics, reproductive history, deciding ideal number of children, knowledge and use of contraceptives were considered. Decision on ideal family size, defined as number of children that woman wants to have in her life, was first assessed by asking if whether woman has decided ideal number of children to have or not and then, if she had decided, number of children she wants to have.

Having information about available contraceptive methods, place where a person can get contraceptives, importance and side effects of contraceptives were considered to assess knowledge about contraception. Women’s perceptions toward sets of statements on benefits (for both woman and family) and side effects of contraception were structured using Likert’s five points scale and used to measure attitude towards contraception.

### Data analysis

Data was entered into Epi-Info vision 7 and managed by using SPSS Version 20 for Windows. Descriptive statistics such as frequency, percentages, mean and standard deviation were used to describe study participants and other study variables. Knowledge and attitude responses were evaluated out of 100 and summarized using quartile. The fourth quartile or scoring 75% and above was categorized as good knowledge or positive attitude. Below these scores were categorized as poor knowledge or negative attitude. Independent variables with *P*-value of less than 0.25 during bivariate binary logistic regression analysis were considered in multivariable binary logistic regression model. Finally, variables with *P*-value of less than 0.05 during multivariable binary logistic regression were reported with adjusted odds ratio (AOR) and 95% CI as factors associated with deciding ideal family size.

### Ethical consideration

Ethical clearance was obtained from the Ethical Review Committee of Regional Health Bureau (Ref. £’6-19-20438). Verbal consent was approved and used for original study. The data used was anonymous and kept confidential.

## Results

### Socio-demographic characteristics of respondents

A total of 3,205 women in the reproductive age group (15–49) were included in this study. Since the data used for this paper was originally collected to assess contraceptive utilization, the socio-demographic characteristics and reproductive history (Table 1) of respondents were previously published along with original study objectives (Endriyas et al., 2017). The mean (SD) age of the respondents was 26.25 ± 7.3 years. About half, 1,668 (52%), of respondents were protestants, near to one-forth, 748 (23.3%), were in the age range between 25–29 years and more than half, 1,810 (56.5%), of respondents were housewives.

**Table 1:** Socio-demographic characteristics and reproductive history of respondents in Southern Ethiopia.

Variable (*n* = 3,205)	Categories	Number	Percent (%)
Age	15–19 years	658	20.5
	20–24 years	532	16.6
	25–29 years	748	23.3
	30–34 years	508	15.8
	35–39 years	390	12.2
	40–44 years	130	4.1
	45–49 years	25	0.8
	I don’t know	214	6.7
Residence	Urban	452	14.1
	Rural agrarian	2,677	83.5
	Rural pastoralist	76	2.4
Educational status	Cannot read and write	1,201	37.5
	Read and write only	177	5.5
	Primary school (1–8)	1,180	36.8
	High school (9–12)	456	14.2
	Certificate and above	94	6.0
Religion	Orthodox	1,030	32.1
	Protestant	1,668	52.0
	Catholic	87	2.7
	Muslim	372	11.6
	No-religion	40	1.3
	Others	8	0.3
Occupation	Housewife	1,810	56.5
	Government employee	130	4.1
	Private employee	58	1.8
	Agrarian/farmer	273	8.5
	Pastoralist	56	1.7
	Merchant	306	9.6
	Unemployed	87	2.7
	Student	481	15.0
	Others	4	0.1
Marital status (*n* = 3,205)	Married and live together	2,376	74.1
	Divorced/widowed/separated	150	4.7
	Single	679	21.2
Age at marriage (*n* = 2,526)	Less than 18 years	1,360	53.8
	18–20 years	496	19.6
	Greater than 20 years	343	13.6
	I don’t know	327	12.9
Number of alive children (*n* = 3,205)	No child	836	26.1
	1–2 children	842	26.3
	3–4 children	751	23.4
	5 and above children	776	24.2
Number of child death (*n* = 3,205)	No child death	2,767	86.3
	1–2 Children	381	11.9
	3–4 Children	48	1.5
	5 and above children	9	0.3
Knowledge about contraception	Poor knowledge	539	16.8
	Good knowledge	2,666	83.2
Attitude towards contraceptive methods	Negative attitude	1,059	33.0
	Positive attitude	2,146	67.0
Current contraceptive utilization	Yes	1,708	53.3
	No	1,497	46.7

### Reproductive history of respondents

About three forth, 2,376 (74.1%), of respondents were married and from those who were married, about half, 1,360 (53.8%), were married before age of 18 years. About one-fourth (26.1%) of respondents had no alive children while 24.2% had five and more alive children. About half (53.3%) of respondents were current users of contraceptives (Table 1).

### Ideal family size decision

**Figure 1:**
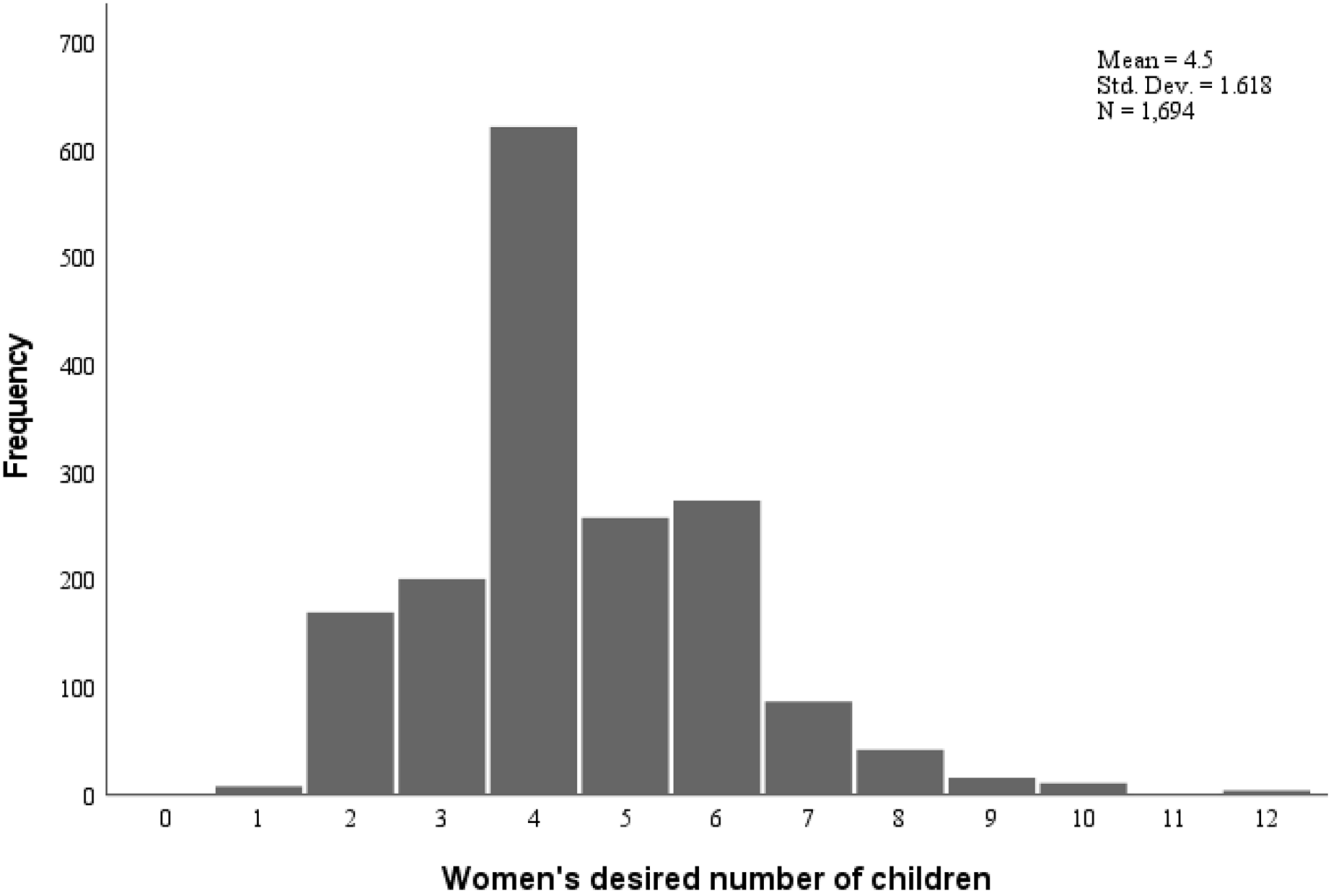
Number of ideal family size desired by women of reproductive age in Southern Ethiopia.

Nearly half, 1,511 (47.1%), of respondents did not decide ideal family size or failed to provide numeric preferences. From those who have decided ideal number of children, 31.2% decide to have one to four children while about one fifth (21.6%) desired to have five or more children. The mean ideal number of children preferred was 4.5 (SD = 1.62). The distribution of ideal family size is presented in Fig. 1. From those who had decided the ideal family size, 511 (30.2%) had already achieved their ideal number of children or more. Regarding urban rural variations, about two-thirds (64.2%) of urban, about half (51.9%) of agrarian and only less than one-fifth (18.4%) of pastoralist respondents decided ideal family size (Table 2).

**Table 2:** Urban rural variation of ideal family size decision among women of reproductive age groups in Southern Ethiopia.

Decided ideal family size	Cluster			Total No (%)
	Urban No (%)	Agrarian No (%)	Pastoralist No (%)	
No	162 (35.8)	1,287 (48.1)	62 (81.6)	1,511 (47.1)
Yes	290 (64.2)	1,390 (51.9)	14 (18.4)	1,694 (52.9)
Total	452 (100)	2,677 (100)	76 (100)	3,205 (100)

### Factors associated with ideal family size decision

In multivariable binary logistic regression analysis at 95% CI (*P* < 0.05), variables associated with deciding numeric ideal family size were place of residence, educational status, age, marital status, number of alive children, overall knowledge about contraceptives and current contraceptive use (Table 3).

**Table 3:** Bivariate and multivariable analysis of factors associated with deciding ideal family size among women of reproductive age groups in Southern Ethiopia.

Variables	Decided ideal family size		COR (95.0% CI)	*P*-value	AOR (95.0 % CI)	*P*-value
	No No (%)	Yes No (%)				
Residence						
Urban	162 (35.8)	290 (64.2)	1			
Agrarian	1,287 (48.1)	1,390 (51.9)	0.60 [0.49–0.74]	<0.001	0.71 [0.57–0.89]	0.03
Pastoralist	62 (81.6)	14 (18.4)	0.13 [0.07–0.23]	<0.001	0.28 [0.14–0.53]	<0.001
Educational status						
No formal education	723 (52.5)	655 (47.5)	1			
Primary school	525 (44.5)	655 (55.5)	1.38 [1.18–1.61]	<0.001	1.52 [1.27–1.82]	<0.001
High school	201 (44.1)	255 (55.9)	1.40 [1.13–1.73]	0.02	1.81 [1.34–2.37]	<0.001
Certificate and above	62 (32.5)	129 (67.5)	2.29 [1.67–3.17]	<0.001	2.24 [1.49–3.36]	<0.001
Age of the respondents						
15–19 years	380 (57.8)	278 (42.2)	1			
20–24 years	204 (38.3)	328 (61.7)	2.20 [1.74–2.78]	<0.001	0.90 [0.66–1.25]	0.54
25–29 years	304 (40.6)	444 (59.4)	1.99 [1.61–2.47]	<0.001	0.73 [0.52–1.03]	0.07
30–34 years	247 (48.6)	261 (51.4)	1.44 [1.14–1.82]	0.002	0.57 [0.39–0.83]	0.003
35–39 years	171 (43.8)	219 (56.2)	1.75 [1.36–2.25]	<0.001	0.69 [0.46–1.03]	0.07
≥ 40 years	92 (59.4)	63 (40.6)	0.94 [0.66–1.34]	0.72	0.44 [0.27–0.71]	<0.001
I don’t know	113 (52.8)	101 (47.2)	1.22 [0.89–1.66]	0.20	0.77 [0.51–1.19]	0.24
Occupation						
Housewife	780 (43.1)	1,030 (56.9)	1			
Employee	64 (34.0)	124 (66.0)	1.47 [1.07–2.01]	0.02	1.26 [0.84–1.88]	0.26
Farmer/agrarian/pastoralist/	193 (58.7)	136 (41.3)	0.53 [0.42–0.68]	<0.001	0.88 [0.67–1.15]	0.35
Merchant/daily laborer/	134 (43.2)	176 (56.8)	0.99 [0.78–1.27]	0.96	1.04 [0.79–1.34]	0.78
Unemployed	48 (55.2)	39 (44.8)	0.61 [0.40–0.95]	0.03	0.94 [0.57–1.55]	0.82
Student	292 (60.7)	189 (39.3)	0.49 [0.40–0.60]	<0.001	0.83 [0.58–1.17]	0.29
Marital status						
Married and live together	998 (42.0)	1,378 (58.0)	1		1	
Single	431 (63.5)	248 (36.5)	0.42 [0.35–0.50]	<0.001	0.56 [0.37–0.82]	0.003
Divorced/widowed/separated	82 (54.7)	68 (45.3)	0.60 [0.43–0.84]	0.003	0.77 [0.54–1.12]	0.17
Number of living children						
No child	503 (60.2)	333 (39.8)	1			
1–2	301 (35.7)	541 (64.3)	2.72 [2.23–3.31]	<0.001	1.47 [1.05–2.08]	0.03
3–4	336 (44.7)	415 (55.3)	1.87 [1.53–2.28]	<0.001	1.30 [0.90–1.88]	0.16
5+	371 (47.8)	405 (52.2)	1.65 [1.35–2.01]	<0.001	1.42 [0.97–2.10]	0.07
Experience of child death						
No	1,301 (47.0)	1,466 (53.0)	1			
Yes	210 (47.9)	228 (52.1)	0.96 [0.79–1.18]	0.72		
Knowledge about contraception						
Poor knowledge	380 (70.5)	159 (29.5)	1			
Good knowledge	1,131 (42.4)	1,535 (57.6)	3.24 [2.65–3.96]	<0.001	1.61 [1.24–2.07]	<0.001
Attitude towards contraception						
Negative attitude	629 (59.4)	430 (40.6)	1			
Positive attitude	882 (41.1)	1,264 (58.9)	2.10 [1.81–2.44]	<0.001	1.20 [0.99–1.45]	0.05
Current contraceptive use						
No	882 (58.9)	615 (41.1)	1			
Yes	629 (36.8)	1,079 (63.2)	2.46 [2.13–2.84]	<0.001	1.50 [1.25–1.81]	<0.001

Women from agrarian and pastoralist areas were 29% and 72% less likely to decide ideal family size as compared to women from urban setting with AOR of 0.71 [0.57–0.89] and 0.28 [0.14–0.53] respectively. Regarding educational status, women who attended primary school, secondary school and college and above were 1.5 times, 1.8 times and 2.2 times more likely to decide ideal family size than women who did not attend formal education.

Concerning knowledge about contraceptive methods and current use, women with good knowledge about contraceptive methods were 1.6 times more likely to decide ideal family size with AOR of 1.61 [1.24–2.07] and current contraceptive users were 1.5 times more likely to decide ideal family size than non-users (AOR 1.50 [1.25–1.81]).

Furthermore, older ages 30–34 years and greater than 40 years were 43% and 56% less likely to decide ideal family size than age groups of 15–19 years with AOR of 0.57 [0.39–0.83] and 0.44 [0.27–0.71] respectively. Single women as compared to those living together and women that did not have children as compared to those who had one-to-two children were less likely to decide family size.

## Discussion

The main findings of this study indicated that about half of respondents did not decide ideal family size or failed to provide numeric preferences. From those who had decided ideal family size, more than one fifth prefer high fertility. The mean ideal number of children preferred was 4.5. Place of residence, educational status, age, marital status, number of alive children, overall knowledge about contraceptives and current contraceptive use were associated with ideal family size decision.

The Universal Declaration of Human Rights states that the choice and decision about the size of family must be made by the family itself (United Nations, 1995). This means all couples and individuals have the right to decide responsibly the number and spacing of their children to have happy family. To achieve this goal, individuals should get information and services that can help them (United Nations, 1995), from which counselling on and providing contraceptive can be mentioned (The Open University, 2017).

The mean ideal family size of this study (4.5) was comparable with that of United Nations estimates of total fertility rate (TFR) for sub-Saharan Africa between 2015–2020 that reported 4.7 births per woman (Bongaarts, 2020). Various and complex factors at individual, familial and societal levels influence child bearing and timing both for women (Benzies et al., 2006) and men (Oiomu, 2000). Different studies (Akram et al., 2020; Harbour, 2011; Dibaba & Mitike, 2016; Matsumoto & Yamabe, 2013) on family size preferences reported that current age, age at marriage, educational status, number of alive children, economic status, knowledge about contraceptives, occupation and current contraceptive use are factors associated with desired family size. The finding of this study is also in line with these reports.

Women from agrarian and pastoralist areas, women who did not attend formal education, older women and women with poor knowledge about contraceptive methods were less likely to decide ideal family size than their reference groups. This might be associated with limited access to information and services, and awareness of respondents because women empowerment enhances fertility decision-making (Atake & Gnakou Ali, 2019; Haque et al., 2021; Bongaarts, 2020). Similarly, current contraceptive users were more likely to decide ideal family size than non-users, which could be due to the awareness created during counselling (Bongaarts, 2020; Feyisetan & Casterline, 2000).

Single women as compared to those living together with partners and women that did not have children as compared to those who had 1–2 children were less likely to decide ideal family size, which could indicate ideal family size decision is assisted by partner (Duvander et al., 2020) because in this study, most of women that had no child were single.

Even though current contraceptive users were 1.5 times more likely to decide ideal family size, 36.8% of this group did not decide ideal family size. In the study setting, contraceptive discontinuation and early removal of long-acting contraceptive methods (removal before six months) are challenges of FP program, which is also wastage of resources. Assisting couples in deciding ideal family size may help them to continue using contraceptives as they could try to achieve their goal. This is because women who have their desired number of children are more likely to use contraceptive methods (Fadeyibi et al., 2022).

### Limitations

As we used secondary data, the study has the following limitations. First, the study lacks some important variables like economic status and significant number of respondents did not give their age that can explain family size decision. Second, due to social desirability bias, some of respondents might have reported desire for small family size. Moreover, the study lacks data on satisfaction of respondents with available number and sex composition of children that have impact on family size decision (Fadeyibi et al., 2022; Suchindran, Ramakumar & Sathi Devi, 1993).

## Conclusion

About half of respondents did not decide ideal numeric family size. For those who have decided ideal number of children, the mean value was comparable with that of Sub-Saharan estimate. About one fifth of those who have decided ideal number of children desire to have five or more children. Less empowered women such as women from rural area, women who did not attend formal education, women who have poor knowledge about contraceptive methods and women who do not use contraceptive methods are less likely to decide ideal family size. Women from such less empowered groups should get information like during counselling for family planning that can empower women to decide ideal family size.

## Supplemental Information

10.7717/peerj.15103/supp-1 Supplemental Information 1 Dataset.

## Lists of Abbreviations

FP: Family Planning
SNNPR: Southern Nations, Nationalities, and People’s Region
SPSS: Statistical Package for the Social Sciences

## Additional Information and Declarations

### Data Availability

The following information was supplied regarding data availability: The raw data is available as a Supplemental File.
